# Elevated Serum Telomerase Level and Peripheral Blood *hTERT* Gene Expression in Patients with Stable Coronary Artery Disease

**DOI:** 10.3390/genes17030276

**Published:** 2026-02-27

**Authors:** Caglar Ozmen, Nihal Inandiklioglu, Omer Tepe, Anıl Akray, Mustafa Gok, Imam Gunay, Abdulkadir Iltas, Pinar Ozmen Yildiz, Hatice Rahimova, Mustafa Demirtas

**Affiliations:** 1 Department of Cardiology, Faculty of Medicine, Çukurova University, 01330 Adana, Türkiye; caglarozm@hotmail.com; 2 Department of Medical Biology, Faculty of Medicine, Yozgat Bozok University, 66100 Yozgat, Türkiye; nihal.inandiklioglu@yobu.edu.tr; 3 Department of Cardiology, Osmaniye State Hospital, 80000 Osmaniye, Türkiye; omer.tepe@windowslive.com; 4 Department of Cardiology, Iskenderun State Hospital, 31200 Hatay, Türkiye; anl_akry@hotmail.com; 5 Department of Cardiology, Kayseri City Hospital, 38080 Kayseri, Türkiye; mustafagok2010@gmail.com; 6 Department of Cardiology, Yozgat City Hospital, 66100 Yozgat, Türkiye; dr.imamgunay@hotmail.com; 7 Department of Cardiology, Ömer Halisdemir Training and Research Hospital, 51100 Niğde, Türkiye; abdulkadiriltas@gmail.com; 8 Department of Cardiology, Adana City Training and Research Hospital, Health Sciences University, 01230 Adana, Türkiye; drmustafademirtas@gmail.com; 9 Department of Cardiology, Darıca Farabi Eğitim Araştırma Hastanesi, 41700 Darıca, Türkiye; rahimli.89@mail.ru

**Keywords:** telomerase, *hTERT*, stable coronary artery disease, gene expression, ELISA

## Abstract

Background/Objectives: Telomeres and telomerase play crucial roles in cellular aging and genome stability. Emerging evidence indicates that alterations in telomerase activity and telomerase reverse transcriptase (*hTERT*) gene expression may be involved in cardiovascular pathophysiology. However, data on telomerase regulation in patients with stable coronary artery disease (CAD) are limited. This study aimed to compare serum telomerase concentration and *hTERT* gene expression levels between patients with stable CAD and healthy controls. Methods: A total of 52 patients diagnosed with stable CAD and 50 age-matched healthy controls were enrolled prospectively. Telomerase concentrations were measured in serum samples using the ELISA method, and *hTERT* mRNA expression was measured in blood samples using RT-PCR. Results: Serum telomerase levels were significantly higher in patients with stable CAD compared with controls (*p* < 0.05). Similarly, *hTERT* gene expression was upregulated in the patient group (*p* < 0.05). Multivariable analysis showed that increased log-transformed telomerase levels (AOR: 2.12, 95% CI: 1.14–5.13, *p* = 0.024) and *hTERT* expression (AOR: 1.79, 95% CI: 1.09–3.27, *p* = 0.037) were independently associated with coronary vessel involvement in stable CAD. These findings indicate an increase in both telomerase level and *hTERT* transcriptional activity in stable CAD. Conclusions: Increased telomerase level and *hTERT* expression may reflect a compensatory response to chronic vascular stress and are associated with disease severity in stable CAD.

## 1. Introduction

Telomeres are heterochromatic regions located at the ends of chromosomes, composed of specific, tandemly repeated DNA sequences. Telomeres progressively shorten with each cell division, and therefore telomere length is considered a marker of aging. Telomerase is a DNA polymerase with reverse transcriptase activity that elongates the ends of telomeric DNA by adding tandemly repeated telomeric sequences [1]. Telomerase reverse transcriptase (abbreviated as hTERT in humans) serves as the catalytic component of telomerase and, in association with the telomerase RNA subunit (TERC), constitutes the core functional element of the telomerase complex [2]. Although telomerase activity is mainly confined to germline, pluripotent embryonic, and hematopoietic progenitor cells, recent studies have also demonstrated its presence in differentiated, non-proliferative somatic cells of the cardiovascular system, suggesting that telomere biology may contribute to the development and progression of cardiovascular diseases [3].

In the Coronary Artery Risk Development in Young Adults (CARDIA) Study, high telomerase activity in leukocytes was associated with a higher prevalence of calcified atherosclerotic plaque, which was highlighted as a marker of plaque progression in individuals with short telomeres [4]. Aging, smoking, psychological stress, obesity, hypertension, diabetes, and treatment, as well as atherosclerotic risk factors, can be attributed to telomere shortening. Okuda et al. [5] observed a negative relationship between telomere length and the degree of atherosclerosis, but this relationship was not consistently significant after adjusting for age. Endothelial changes in telomere shortening may play a role in atherogenesis by increasing proinflammatory reactions and promoting the proliferation of highly unstable atherosclerotic plaques [6]. It is known that chronic cardiovascular disorders arise from interconnected molecular networks in which oxidative stress and inflammation function as central regulators [7]. Furthermore, higher leukocyte telomerase activity was associated with a higher prevalence of calcified atherosclerotic plaques, and this relationship was stronger in those with short telomeres [4].

Given the limited data on telomerase biology in patients with stable coronary artery disease (CAD), this study was designed to test the prespecified hypothesis that telomerase concentration and *hTERT* gene expression are significantly altered in patients with stable CAD compared with controls, and that these molecular markers are associated with disease presence and severity.

## 2. Materials and Methods

### 2.1. Study Population

A total of 52 consecutive patients between the ages of 50 and 70 years, diagnosed with stable coronary artery disease (CAD), were prospectively included in this study. All participants underwent elective coronary angiography, with or without percutaneous coronary intervention (PCI), at Çukurova University Hospital, Department of Cardiology. Only patients whose coronary lesions were confirmed by coronary angiography (identified stenosis > 50%) were eligible for the study. The decision to perform PCI was made by the attending physicians based on the patients’ clinical presentation and angiographic findings. The severity of CAD was evaluated according to the number of affected vessels and categorized as one-, two-, or three-vessel disease. The exclusion criteria were as follows: (1) if the patient was not willing to participate; (2) if the patient was diagnosed with acute coronary syndrome (ACS), cancer and active liver, lung, or neuromuscular system diseases were excluded from the study. The control group consisted of 50 age-matched low-risk healthy individuals with no history of any disease were selected from healthy people at Çukurova University Hospital Cardiology Department who visited for their regular checkups. However, unlike the patient group, these individuals did not undergo invasive coronary angiography; therefore, the presence of subclinical atherosclerosis cannot be entirely ruled out. These study groups data were collected from patient’s and control’s medical records. The study was conducted in accordance with the Helsinki Declaration, and informed consent was obtained from all participants. The study was approved by the Çukurova University Ethics Committee (protocol code 89 and approval date 14 June 2019). In accordance with STROBE-ME (Strengthening the Reporting of Observational Studies in Epidemiology—Molecular Epidemiology) recommendations, we explicitly state that all biospecimens (serum and peripheral blood) were collected specifically for this research study following prospective enrollment and were not derived from routine clinical sampling [8]. A completed STROBE-ME checklist, indicating the page numbers where each required reporting item is addressed, is provided as Supplementary Materials.

### 2.2. RT-PCR Analysis

To avoid the confounding effects of periprocedural stress and inflammation, peripheral blood samples were systematically collected from all patients in the fasting state prior to elective coronary angiography or PCI. Total RNA was isolated from peripheral blood samples within 2 h of collection using the High Pure RNA Isolation Kit (catalog no: 11828665001, Roche, Mannheim, Germany) for expression analysis. RNA samples were then converted to cDNA using the Transcriptor First Strand cDNA Synthesis Kit (catalog no: 04896866001, Roche, Mannheim, Germany). cDNA samples were stored at −20 °C for a maximum of 3 months until analysis. cDNA concentrations were measured by fluorometer (QFX, 3411 Silverside Road, 100 Hagley Building, Wilmington, DE 19810, USA) and all samples were standardized to a concentration of 100 ng/μL. hTERT (Thermo, Waltham, MA, USA, catalog no: Hs00972650_m1) gene expression was analyzed using RT-PCR. The beta-actin (ACTB) gene was used as a control gene. cDNA samples were examined three times under the same conditions, and the average of these three measurements was used. The quantification cycle (Ct) was recorded for each sample, and relative expression levels were analyzed using the 2^−ΔΔCt^ method. 

### 2.3. ELISA Analysis

Fasting venous blood samples were collected in the morning between 7:00 and 8:00 a.m. The samples were drawn into serum-separating vacuum tubes and centrifuged at 3000 rpm for 10 min to obtain serum. The resulting serum samples were stored at −20 °C until analysis. Serum telomerase levels were quantified using a commercially available ELISA kit (Cat. No. E0934Hu, Human Telomerase ELISA Kit, Bioassay Technology Laboratory, Zhejiang, China), with a measurement range of 0.5–150 IU/mL. Optical density values for both samples and standards were measured at 450 nm using a Thermo Scientific Multiskan GO microplate reader (Thermo Fisher Scientific Oy, Ratastie, Finland). Results were expressed in IU/mL. The serum samples were studied three times under the same conditions. The mean of these two measurements was used in the analyses. To maintain methodological rigor, all biochemical evaluations were performed by personnel blinded to the clinical and angiographic status of the study participants.

### 2.4. Statistical Analysis

All statistical evaluations were carried out using SPSS (version 20, SPSS Inc., Chicago, IL, USA). The distributional characteristics of continuous variables were examined with the Kolmogorov–Smirnov and Shapiro–Wilk normality tests. Differences in categorical variables across groups were assessed using either the Chi-square test or Fisher’s exact test, as appropriate. Comparisons between groups were conducted using Student’s *t*-test for normally distributed data and the Mann–Whitney U test for non-normally distributed data. A multivariate analysis was performed using an ordinal logistic regression analysis for independent variables that were related to the number of stenosed vessels in stable CAD patients. The proportional odds (parallel lines) assumption was assessed using the Brant test and was found not to be violated. Continuous variables with skewed distributions, including telomerase levels measured by ELISA and *hTERT* gene expression levels, were log-transformed prior to inclusion in the regression models. A *p*-value of less than 0.05 was considered statistically significant.

## 3. Results

**Figure 1:**
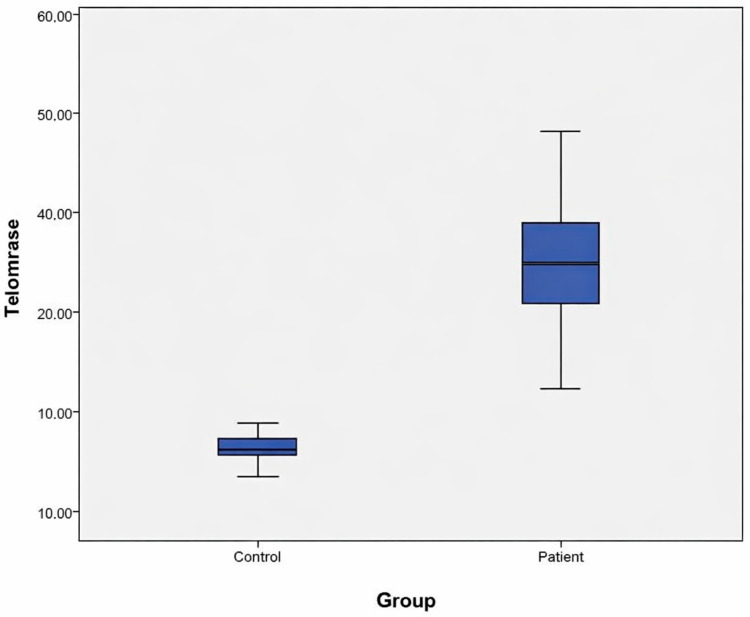
Distribution of serum telomerase levels between the patient group (33.51 IU/mL) and the control group (17.08 IU/mL).

The demographic characteristics, laboratory findings, and ELISA results of the study groups are presented in Table 1 and Figure 1. Statistical comparisons between the patient and control groups revealed significant differences in hsCRP levels, left atrial size, and ejection fraction (*p* < 0.05). However, no statistically significant differences were observed between the groups for the remaining parameters (*p* > 0.05). Regarding serum telomerase levels, the patient group exhibited significantly higher concentrations compared with the control group (*p* < 0.05) (Table 1, Figure 1).

**Figure 2:**
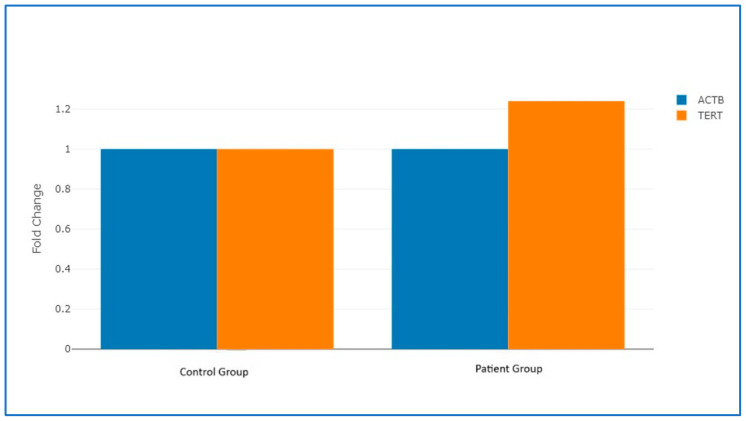
Change in *hTERT* gene 2^ΔCT value in the patient group and the control group. In the patient group, hTERT expression levels were increased by fold change 1.24, compared to the control group.

Evaluation of the *hTERT* gene expression analysis revealed that *hTERT* expression levels were increased in the patient group compared with the control group (fold change: 1.24, *p* = 0.02). The results of this analysis are presented in Table 2 and Figure 2.

In the stable CAD group, multivariable ordinal logistic regression analysis showed that higher log-transformed ELISA telomerase levels (AOR: 2.12, 95% CI: 1.14–5.13, *p* = 0.024) and log-transformed *hTERT* gene expression (AOR: 1.79, 95% CI: 1.09–3.27, *p* = 0.037) were independently associated with an increased number of diseased vessels. Smoking and diabetes mellitus were also independently associated with greater vessel involvement. In contrast, body mass index, hypertension, and hyperlipidemia were not significantly associated with the number of diseased vessels (all *p* > 0.05) (Table 3).

## 4. Discussion

In this study, we compared telomerase concentration and expression between patients with stable CAD and controls. Our findings showed that both serum telomerase level and *hTERT* gene expression were higher in the stable CAD group. In addition, an independent association was found between serum telomerase levels and number of diseased vessels.

While there is epidemiological and genetic evidence linking telomere length and telomerase activity to cardiovascular disease risk, the causal direction and magnitude of the associations remain unclear due to the influence of various factors. Furthermore, genetic manipulations demonstrating the efficacy of *hTERT* gene therapy in pulmonary fibrosis, improving ventricular function, and reducing infarct scarring after acute myocardial infarction have kept alive the hypothesis that *hTERT* may be a potential therapeutic target for cardiovascular disease [9,10]. Telomerase shows measurable levels within the human coronary artery, and this activity is enhanced during the onset and progression of atherosclerosis [11]. *hTERT* is expressed in all cell types, including macrophages, during the atherosclerotic disease process [12,13]. However, the mechanisms underlying telomerase activation in atherosclerotic disease remain incompletely understood.

When examining the limited number of studies in stable CAD patients in the literature, Narducci et al. evaluated 20 unstable angina (UA) and 6 stable angina (SA) patients undergoing percutaneous coronary intervention and found high telomerase activity in polymorphonuclear neutrophils obtained from coronary plaques of UA patients. However, such activity was not observed in patients with SA or in polymorphonuclear neutrophils obtained from peripheral blood [14]. In a study conducted in Turkey, serum telomerase concentrations were not significant in patients with ACS and stable CAD compared to controls. *hTERT* gene expression was found to be lower in patients with ACS and stable CAD compared to controls [15]. Conversely, another study analyzed significantly higher telomerase levels in 22 SA cases, 93 STEMI cases, 7 non-obstructive coronary artery disease (MINOCA), and 6 blood vessel rupture cases compared to controls. Significantly shorter telomeres were found in the other three groups compared to the control and SA groups, and the protective score was also higher in the SA group compared to all other groups [16]. In our previous studies, we found short telomere length in both MINOCA and STEMI patients [17,18]. In interpreting our findings, it is essential to consider the methodological heterogeneity across studies. Previous research by Liu et al. identified telomerase reactivation in 70% of atherosclerotic arteries using the TRAP assay on tissue segments, whereas our study utilized serum ELISA to quantify systemic levels [19]. TRAP measures functional enzyme activity, while ELISA quantifies protein concentration; this may explain discrepancies with studies like Kilinc et al., who found no significant difference in serum levels among Turkish ACS/SCAD populations [15]. Additionally, the use of peripheral blood leukocytes for mRNA expression in our study captures the systemic inflammatory milieu of stable CAD, which differs from studies focusing on local plaque-derived cells. In our study, *hTERT* expression in patients with stable CAD showed a modest but statistically significant 1.24-fold increase compared to controls. Although the precise role of telomerase activation in cardiovascular disease remains unclear, increased telomerase level may reflect a compensatory or adaptive cellular response to chronic vascular stress. Furthermore, although this magnitude of change may seem subtle, it is important to consider that *hTERT* is the rate-limiting component for telomerase function.

The association between serum telomerase levels and the severity of CAD was further investigated in patients with CAD [20]. Given that the natural course of CAD is substantially influenced by the number of affected coronary artery branches, disease severity was stratified according to the number of involved vessels and examined in relation to elevated serum telomerase levels. As shown in Table 3, higher log-transformed serum telomerase levels were independently associated with an increased number of diseased vessels after adjustment for established cardiovascular risk factors. The baseline differences in hsCRP and ejection fraction (EF) between our study groups highlight the complex inflammatory and functional milieu of stable CAD. While these factors could potentially confound the expression of cellular aging markers, our multivariable ordinal logistic regression model demonstrates that log-transformed telomerase levels and hTERT expression remain independently associated with the anatomical extent of the disease (vessel involvement). In addition, increased *hTERT* gene expression, smoking status, and diabetes mellitus were also independently associated with greater disease extent, whereas age, body mass index, hypertension, and hyperlipidemia were not significant predictors. Collectively, these findings suggest that there may be an association between telomerase-related biomarkers and the anatomical severity of coronary artery disease. A study has demonstrated that vessels containing atherosclerotic plaques exhibit significantly shorter telomeres and that telomere shortening is closely associated with the progression and severity of atherosclerosis [21]. In this context, the present results extend existing evidence by suggesting that telomerase activity, in addition to telomere length, may be linked to disease burden in stable CAD. On one hand, the upregulation of telomerase in patients with more severe vessel involvement may represent an adaptive compensatory effort to stabilize DNA ends in response to the accelerated telomere attrition driven by chronic oxidative stress and inflammation in CAD. On the other hand, telomerase is known to possess non-canonical, telomere-independent functions, such as the regulation of reactive oxygen species (ROS) and DNA repair pathways [22,23]. Kurz et al., which demonstrated that chronic oxidative stress accelerates endothelial senescence by doubling the rate of telomere attrition and compromising telomere integrity [6]. Matthews et al. to highlight that vascular smooth muscle cells in human atherosclerosis undergo telomere-based senescence specifically driven by oxidative DNA damage [21]. These help explain why we found a correlation between our biomarkers and the anatomical severity/number of involved vessels, as these markers reflect the cumulative burden of ROS and DNA damage in the vessel wall. Our results indicate that elevated serum telomerase level and hTERT expression are independently associated with the number of involved coronary vessels in patients with stable CAD. However, the cross-sectional observational design of this study precludes the establishment of a formal causal relationship between these molecular markers and the progression of atherosclerosis. Telomere length (TL) data, it remains possible that the observed high telomerase levels are a pathogenic byproduct of a systemic inflammatory environment or increased leukocyte turnover, rather than a successful effort to maintain cellular longevity. Future research integrating both TL and enzymatic markers is essential to clarify whether this molecular surge effectively counteracts vascular aging or merely mirrors the disease’s biological burden.

Gene expression profiling revealed that proapoptotic genes (BAX, CASP1, FAS, FASL, FOS, NFκB2, P53, and PCNA) were markedly upregulated in plaques obtained from patients with non-ST-elevation acute coronary syndrome (ACS), whereas antiapoptotic genes (MDM2, TERT, and XRCC1) showed higher transcriptional activity in plaques from subjects with stable angina (SA) [24]. Although both gene categories were expressed in each condition, the predominance of proapoptotic gene activation was evident in ACS plaques, while antiapoptotic transcripts were more abundant in SA plaques. These findings support the notion that ACS-associated plaques are predisposed to apoptosis through a coordinated regulatory network of gene activation and repression, whereas SA plaques appear to preserve cellular homeostasis and repair functions. Beyond its well-established role in telomere maintenance, telomerase has also been implicated in multiple cellular pathways, including oxidative stress responses, DNA repair, and apoptotic regulation [25]. Enhanced telomerase activity, therefore, not only stabilizes telomere length but also contributes to sustaining cellular integrity and long-term immune competence [26]. Animal studies have reported that telomerase deficiency is associated with an increased risk of cardiovascular disease, impaired glucose metabolism, and insulin secretion [27,28]. Smoking is a significant risk factor for aging-related diseases and increases telomere shortening [29]. Furthermore, in adult comparisons, higher telomerase levels and longer telomere length were found in women compared to men [30]. Accordingly, variations in telomerase levels and *hTERT* expression across studies may be influenced by demographic characteristics, comorbid conditions, and environmental exposures. Finally, the synergy between molecular biology and advanced multimodality imaging is essential for comprehensive risk assessment. Specifically, Cardiac Magnetic Resonance (CMR) provides high-resolution tissue characterization without ionizing radiation. Techniques such as Late Gadolinium Enhancement (LGE) are critical for identifying myocardial scars and assessing myocardial viability or hibernation, which directly informs revascularization decisions and improves risk stratification in patients with established CAD [31]. Combining biochemical and genetic markers, such as hTERT expression, with imaging-derived parameters like ischemia or scar burden, creates a robust roadmap for personalized cardiovascular medicine.

This study has several limitations. First, the sample size was relatively small and drawn from a single center, which may limit the generalizability of the findings. Second, although key confounding variables were considered, unmeasured factors such as detailed dietary habits, physical activity levels, psychosocial stress, and lifelong exposure to environmental risk factors may have influenced telomerase concentration and *hTERT* expression. Third, telomere length was not evaluated together with telomerase levels, which could have provided a more comprehensive understanding of telomere biology in stable CAD. A methodological limitation is that serum telomerase was quantified using ELISA, which measures protein concentration rather than functional enzymatic activity (typically assessed via TRAP assay). Additionally, telomerase levels and *hTERT* expression were measured at a single time point. The limited number of patients (*n* = 52) relative to the number of covariates in our models increases the potential for statistical overfitting, which may affect the stability and precision of the estimated odds ratios. While our sensitivity analyses confirm the stability of these markers, the results should be interpreted as independent associations. Consequently, these molecular markers should be viewed as promising indicators of disease severity that require validation in larger, multicenter prospective cohorts to confirm their predictive utility and clinical robustness.

## 5. Conclusions

In conclusion, serum telomerase concentration and *hTERT* gene expression were significantly higher in patients with stable CAD than in healthy controls and were independently associated with greater disease severity, as reflected by the number of affected coronary vessels. These findings suggest a potential link between telomerase activation and the extent of coronary atherosclerosis, although the underlying mechanisms remain unclear. While these findings suggest that the telomerase system may be a surrogate indicator of anatomical disease severity, they must be interpreted as associative in nature. It remains possible that the observed upregulation is a secondary response to chronic vascular stress (reverse causation) rather than a primary driver of the disease course. Future longitudinal studies are required to determine whether these changes possess predictive value for disease development or clinical outcomes over time.

## Figures and Tables

**Table 1:** Demographic characteristics of the study population.

	Stable CAD (*n* = 52)	Control (*n* = 50)	*p*
Age (year)	55.3 ± 6.7	53.0 ± 6.5	0.092
Sex (M/F)	29/23	27/23	0.379
BMI (kg/m^2^)	26.0 ± 5.4	27.6 ± 6.1	0.677
Heart rate (beat/min)	81.7 ± 16.5	74.3 ± 14.2	0.258
Current/ex-smoker (*n*)	28	21	0.526
Diabetes mellitus (*n*)	21	0	-
Hypertension (*n*)	33	0	-
Hyperlipidemia (*n*)	4	0	-
Previous myocardial infarction (%)	9.6	0	-
**Blood results**			
Haemoglobin (g/dL)	11.6 ± 1.9	12.4 ± 1.6	0.280
Serum Creatinine (mg/dL)	1.1 ± 0.8	1.0 ± 0.6	0.994
Uric acid (mg/dL)	7.2 ± 1.7	6.7 ± 1.7	0.455
hsCRP (mg/L)	1.30 ± 2.14	0.63 ± 1.09	0.023 *
LDL cholesterol (mg/dL)	131.4 ± 25.1	110.2 ± 30.3	0.164
HDL cholesterol (mg/dL)	40.3 ± 8.7	38.1 ± 11.2	0.537
Triglycerides(mg/dL)	183.2 ± 65.1	115.9 ± 81.5	0.185
**Echocardiographic findings**			
Left atrium (mm)	39.2 ± 5.5	37.0 ± 3.2	0.046 *
EF (%)	46.4 ± 8.8	58.7 ± 5.9	0.031 *
LVEDD (mm)	47.5 ± 8.1	44.7 ± 7.5	0.326
LVM (gr)	269.5 ± 54.2	258.7 ± 76.7	0.662
**Coronary angiography**			
1-vessel disease, %	38.5	-	-
2-vessel disease, %	34.6	-	-
3-vessel disease, %	26.9	-	-
Left main trunk disease %	9.6	-	-
**ELISA Telomerase (IU/mL)**	33.51 ± 5.22	17.08 ± 1.33	0.000 *

BMI: body mass index; EF: ejection fraction; HDL: high-density lipoprotein cholesterol; hsCRP: high- sensitive c-reactive protein; LDL: low-density lipoprotein cholesterol; LVEDD: left ventricular end-diastolic diameter; LVM: left ventricular mass; * Data with *p* < 0.05 are highlighted in bold.

**Table 2:** Changes in *hTERT* gene 2^ΔCT and fold change values according to patient and control groups.

Gene Symbol	2^ΔC_T_		Fold Change *	
	Control	Stable CAD	Stable CAD/Control	*p* Value **
***ACTB***	1.00	1.00	1.0	nan
***hTERT***	0.0626	0.0773	1.24	0.02

* Fold-Change (2^−ΔΔCT^) is the normalized gene expression in Test Sample (2(−ΔCT)) divided by the normalized gene expression in Control Sample (2(−ΔCT)). ** *p* values are calculated according to Student’s *t*-test of repeated 2(−ΔCT) values for each gene in the control group and treatment groups, and *p* values less than 0.05 are shown.

**Table 3:** Multivariate ordinal logistic regression analysis regarding the increase in the number of disease vessels in stable CAD group.

Independent Variables	AOR	95% CI	*p* Value
LogELISA Telomerase	2.12	1.14–5.13	0.021 *
Log*hTERT* gene expression	1.79	1.09–3.27	0.037 *
BMI	1.04	0.96–1.15	0.33
Current/ex-smoker	2.52	1.91–7.27	0.041 *
Diabetes mellitus	3.04	1.27–7.63	0.029 *
Hypertension	2.21	0.83–9.1	0.18
Hyperlipidemia	1.03	0.92–1.07	0.32

Multiple ordinal regression was used to identify the factors associated with the increase in the number of diseased vessels, and the results are reported as AORs with 95% CIs. AOR: Adjusted odds ratio; BMI: body mass index; CI, confidence interval; hTERT: telomerase reverse transcriptase. * Data with *p* < 0.05 are highlighted in bold.

## Data Availability

The data presented in this study are available on request from the corresponding author.

## Supplementary Materials

The following supporting information can be downloaded at: https://www.mdpi.com/article/10.3390/genes17030276/s1, Supplementary File: Completed STROBE-ME checklist with page and paragraph references for the reported molecular epidemiological data.
