# Supplementary material for: Elevated Serum Telomerase Level and Peripheral Blood hTERT Gene Expression in Patients with Stable Coronary Artery Disease
Source: Genes (Basel). 2026 Feb 27;17(3):276. doi: 10.3390/genes17030276 (PMC13025165; doi:10.3390/genes17030276)
Supplement: Supplementary file 1 [file genes-17-00276-s001.zip › genes-4163122-supplementary.pdf]

STROBE Statement—Checklist of items that should be included in reports of ***case-control studies***

	Item No	Recommendation
**Title and abstract**	1	(*a*) Indicate the study's design with a commonly used term in the title or the abstract<br>Study design stated in title and abstract- Title page, Abstract
		(*b*) Provide in the abstract an informative and balanced summary of what was done and what was found<br>Balanced summary of methods and results- Abstract
**Introduction**		
Background/rationale	2	Explain the scientific background and rationale for the investigation being reported<br>Scientific background on telomeres and CAD- Introduction, p.1–2
Objectives	3	State specific objectives, including any prespecified hypotheses<br>Prespecified hypothesis stated- End of Introduction, p.2
**Methods**		
Study design	4	Present key elements of study design early in the paper<br>Prospective observational case–control study- Methods 2.1, p.2
Setting	5	Describe the setting, locations, and relevant dates, including periods of recruitment, exposure, follow-up, and data collection<br>Single-center cardiology department, Turkey- Methods 2.1, p.2
Participants	6	(*a*) Give the eligibility criteria, and the sources and methods of case ascertainment and control selection. Give the rationale for the choice of cases and controls<br>Eligibility criteria and selection of cases/controls- Methods 2.1, p.2–3
		(*b*) For matched studies, give matching criteria and the number of controls per case
Variables	7	Clearly define all outcomes, exposures, predictors, potential confounders, and effect modifiers. Give diagnostic criteria, if applicable<br>Definition of outcomes, exposures, confounders- Methods 2.2–2.4, p.3–4
Data sources/ measurement	8*	For each variable of interest, give sources of data and details of methods of assessment (measurement). Describe comparability of assessment methods if there is more than one group<br>RT-PCR and ELISA protocols described- Methods 2.2–2.3, p.3–4
Bias	9	Describe any efforts to address potential sources of bias<br>Blinded laboratory analysis, exclusion criteria- Methods 2.1–2.3, p.3–4
Study size	10	Explain how the study size was arrived at<br>Total sample size reported- Methods 2.1, p.2
Quantitative variables	11	Explain how quantitative variables were handled in the analyses. If applicable, describe which groupings were chosen and why<br>Log-transformation and fold-change methods- Methods 2.4, p.4
Statistical methods	12	(*a*) Describe all statistical methods, including those used to control for confounding<br>Regression and group comparisons- Methods 2.4, p.4
		(*b*) Describe any methods used to examine subgroups and interactions<br>Vessel number stratification- Results, p.6–7
		(*c*) Explain how missing data were addressed<br>No missing data reported- Results, p.5
		(*d*) If applicable, explain how matching of cases and controls was addressed
		(*e*) Describe any sensitivity analyses
**Results**		
Participants	13*	(a) Report numbers of individuals at each stage of study—eg numbers potentially

		eligible, examined for eligibility, confirmed eligible, included in the study, completing follow-up, and analysed<br>Numbers analyzed in each group- Results, p.5
		(b) Give reasons for non-participation at each stage
		(c) Consider use of a flow diagram
Descriptive data	14*	(a) Give characteristics of study participants (eg demographic, clinical, social) and information on exposures and potential confounders<br>Demographic and clinical characteristics- Results Table 1, p.5
		(b) Indicate number of participants with missing data for each variable of interest
Outcome data	15*	Report numbers in each exposure category, or summary measures of exposure<br>Telomerase levels and hTERT expression- Results Table 2, p.6
Main results	16	(*a*) Give unadjusted estimates and, if applicable, confounder-adjusted estimates and their precision (eg, 95% confidence interval). Make clear which confounders were adjusted for and why they were included<br>Adjusted odds ratios with CI- Results Table 3, p.7
		(*b*) Report category boundaries when continuous variables were categorized
		(*c*) If relevant, consider translating estimates of relative risk into absolute risk for a meaningful time period
Other analyses	17	Report other analyses done—eg analyses of subgroups and interactions, and sensitivity analyses<br>Ordinal regression by vessel number- Results, p.6–7
**Discussion**		
Key results	18	Summarise key results with reference to study objectives<br>Summary linked to objectives- Discussion, p.7–8
Limitations	19	Discuss limitations of the study, taking into account sources of potential bias or imprecision. Discuss both direction and magnitude of any potential bias<br>Sample size, cross-sectional design- Discussion, p.9
Interpretation	20	Give a cautious overall interpretation of results considering objectives, limitations, multiplicity of analyses, results from similar studies, and other relevant evidence<br>Comparison with literature, biological meaning- Discussion, p.7–9
Generalisability	21	Discuss the generalisability (external validity) of the study results<br>Single-center limitation discussed- Discussion, p.9
**Other information**		
Funding	22	Give the source of funding and the role of the funders for the present study and, if applicable, for the original study on which the present article is based<br>Funding source reported-Funding section, p.10

*Give information separately for cases and controls.

Note: An Explanation and Elaboration article discusses each checklist item and gives methodological background and published examples of transparent reporting. The STROBE checklist is best used in conjunction with this article (freely available on the Web sites of PLoS Medicine at http://www.plosmedicine.org/, Annals of Internal Medicine at http://www.annals.org/, and Epidemiology at http://www.epidem.com/). Information on the STROBE Initiative is available at http://www.strobe-statement.org.
